# Effects of Combined Salt and Heat Stress on Agronomic Traits, Photosynthetic Parameters, and Physiological Biochemistry in Six Alfalfa (*Medicago sativa* L.) Cultivars

**DOI:** 10.3390/plants14162479

**Published:** 2025-08-10

**Authors:** Lihe Su, Rongzheng Huang, Dongqing Fu, Yongcheng Chen, Xudong Zhang, Ying Chen, Chaorong Liu, Tianyu Hu, Chunhui Ma

**Affiliations:** College of Animal Science and Technology, Shihezi University, Shihezi 832000, China; lihesu2025@163.com (L.S.); huangrz2013@163.com (R.H.); fudongqing2024@163.com (D.F.); chenyonchn@163.com (Y.C.); zxd13519972930@126.com (X.Z.); yingchen520@126.com (Y.C.); chaorongliu@126.com (C.L.); hty0314@163.com (T.H.)

**Keywords:** *Medicago sativa*, combined salt and heat stress, photosynthesis, plant growth

## Abstract

Climate change due to global warming increases the susceptibility of plants to multiple combined stresses. Soil salinization and high temperature stresses that co-occur in arid/semiarid regions severely restrict the growth and development of plants. Although alfalfa (*Medicago sativa* L.) is an important forage grass, the physiological mechanisms driving its responses to combined salt and heat stress are not yet clear. This study aimed to reveal the physiological and biochemical response mechanisms of six alfalfa cultivars to different stresses by comparing plant morphology, agronomic traits, photosynthetic characteristics, and physiological and biochemical responses under control conditions, salt stress (200 mM NaCl), heat stress (38 °C), and combined salt and heat stress. Compared with single stresses, combined stress significantly inhibited the growth and biomass accumulation of alfalfa. Under combined stress, the cultivars presented decreases in plant height and total fresh biomass of 11.87–26.49% and 28.22–39.97%, respectively, compared with those of the control plants. Heat stress promoted alfalfa photosynthesis by increasing stomatal conductance, net photosynthetic rate, and transpiration rate, while salt stress and combined stress significantly suppressed these effects. Combined stress significantly increased the concentration of Na^+^ but decreased that of K^+^ and the relative water content in alfalfa leaves. Compared with the control and single stress treatments, combined stress significantly increased the level of membrane lipid peroxidation and accumulation of reactive oxygen species. The proline contents in the leaves of the different alfalfa cultivars were 2.79–11.26 times greater under combined stress than in the control. Combined stress causes alfalfa to redistribute energy from growth and development to stress defense pathways, ultimately leading to a reduction in biomass. Our study provides theoretical guidance for analyzing the mechanisms of grass resistance to combined salt and heat stress.

## 1. Introduction

In recent years, climate change caused by global warming has led to increases in the frequency and intensity of extreme weather events, such as droughts, high temperatures, and cold snaps [1]. These climate conditions interact with harsh soil conditions (e.g., soil salinity and nutrient deficiency), increasing plant exposure to multiple stresses [2]. Plants can adapt to individual abiotic stresses under natural conditions by regulating their physiological and metabolic mechanisms [3]. However, when multiple abiotic stresses occur simultaneously, they not only seriously affect the growth and reproduction of plants but also interfere with the interactions between plants and other organisms, posing a major threat to the sustainable development of agriculture and animal husbandry [4]. Therefore, understanding the interactions among a combination of stressors and their impact mechanisms on plant growth and survival is crucial for improving agricultural and livestock production.

Various mechanisms underlying plant responses to multiple abiotic stresses have been reported. When plants are under drought or salt stress, the stomata are usually closed to prevent leaf dehydration, while under heat stress, the stomata are kept open to cool the leaves by transpiration [5,6,7]. However, when drought and heat stress occur simultaneously, plants typically close their stomata to restrict transpiration; this response is influenced by the intensity and timing of the stress [8]. Notably, new findings reveal that soybean has evolved a unique ‘differential transpiration’ strategy under combined drought and heat stress, where stomata on the leaves close while stomata on the pods and flowers (sepals) remain open to sustain transpiration cooling, effectively preventing thermal damage to the reproductive organs [9,10]. Individual low-intensity stresses (e.g., acidity, metal exposure, high light, heat, salinity, and oxidation) have minimal effects on the growth of plants such as *Arabidopsis thaliana*, rice, and maize. However, when these stresses occur in combination, they severely inhibit plant growth and survival [11,12].

Global warming has led to increasingly frequent extreme heat events, while global soil salinization is becoming increasingly severe, resulting in crop yield losses [13,14,15]. Heat stress not only damages the chloroplast structure of plants but also triggers excessive accumulation of reactive oxygen species (ROS), causing irreversible cellular oxidative damage [16]. Under salt stress, plant roots absorb excessive harmful ions such as Na^+^ and Cl^−^, which interfere with cellular growth and metabolic processes, significantly reducing plant productivity [17,18,19]. Under field conditions, crops are highly susceptible to the combined effects of soil salinization and high temperatures. Combined salt and heat stress significantly inhibits plant photosynthesis, especially by affecting the carbon assimilation process and consequently reducing crop productivity [20]. The level of salinity is a critical factor determining the response pattern of plant stomata; when low salinity is combined with high temperature, the stomatal conductance is essentially not affected, whereas combined high-salinity and high-temperature stress significantly inhibits the stomatal conductance and induces the accumulation of ROS [21]. When the number of stress factors increases to three (salt, heat, and drought), crops such as wheat, barley, and quinoa exhibit more severe growth inhibition and yield loss than they do under a single stress. Heat and drought even exacerbate the absorption of Na^+^ by plant roots, resulting in severe disruption of ion homeostasis. Moreover, crops synthesize osmotic regulators such as proline to enhance stress resistance [22,23,24]. Li et al. [25] further reported that heat stress was the dominant stressor for tomato plants under combined salt and heat stress and that the oxidative phosphorylation pathway plays a key role in the response of tomato plants to combined stress. However, studies have also revealed the specific adaptation mechanisms of plants to combined stresses. Studies on tomatoes have shown that the combination of salt and heat stress can alleviate the damage caused by salinity to plants, which could be explained by the accumulation of osmotic regulatory substances that maintain a higher K^+^ content and lower Na^+^/K^+^ ratio in leaves under combined stress [26]. These studies provide important insights into the mechanism of combined salt and heat stress in plants.

Alfalfa (*Medicago sativa* L.), a perennial legume, is considered one of the most important forages worldwide because of its high nutritional quality and wide adaptability, and its high yield is beneficial for agricultural production. Alfalfa is tolerant to abiotic stresses, such as salinity, low temperature, heat, and drought [27,28,29,30]. However, most research has focused only on the response of alfalfa to a single stress, and comprehensive research on the combined effects of salt and heat stress on alfalfa is lacking. Therefore, our study aimed to (1) explore the effects of salt stress, heat stress, and combined stress on the phenotype, growth, and biomass of six alfalfa cultivars and (2) evaluate the photosynthetic parameters and physiological and biochemical tolerance mechanisms under salinity, heat, and combined stress in six alfalfa cultivars.

## 2. Results

### 2.1. Effects of Single and Combined Stresses on the Morphology and Growth Parameters of Alfalfa Cultivars

**Figure 1:**
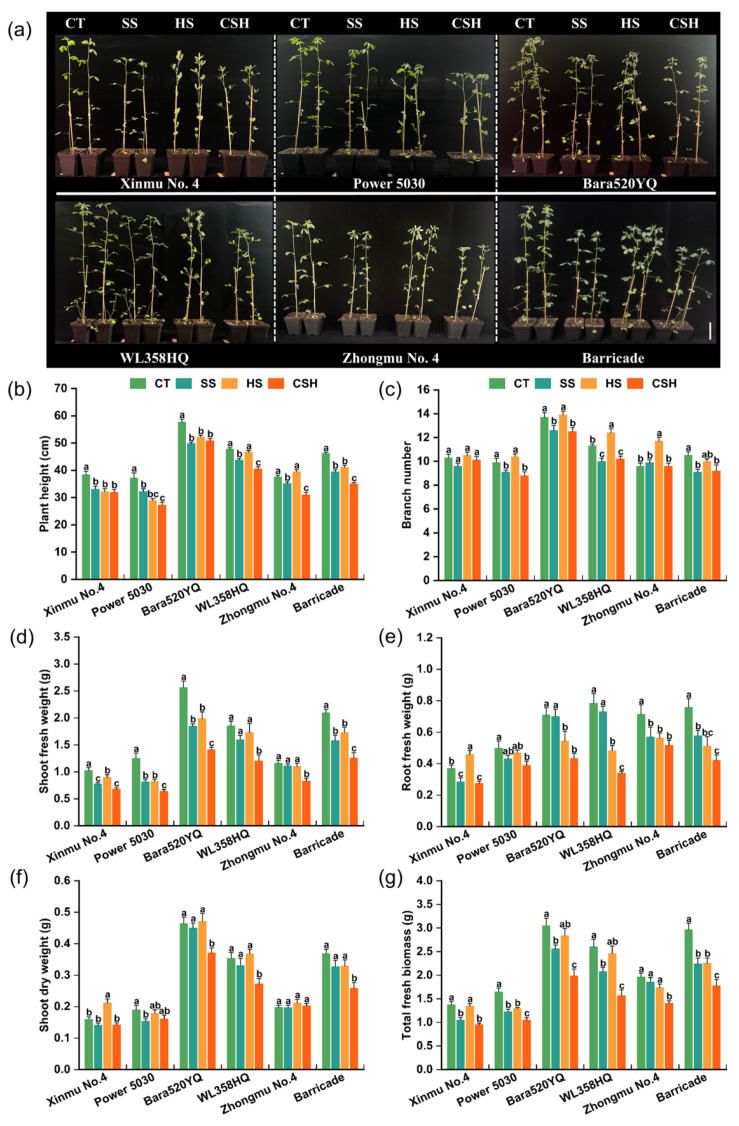
Changes in the phenotypes and agronomic traits of different alfalfa cultivars under single and combined stresses. (**a**), CT, SS, HS, and CSH represent the control, salt stress, heat stress, and combined stress, respectively; the same applies in the figures below. (**b**) Plant height, (**c**) branch number, (**d**) shoot fresh weight, (**e**) root fresh weight, (**f**) shoot dry weight, and (**g**) total fresh weight. Data are presented as the mean ± standard error (*n* = 10). Significant differences at the *p* < 0.05 level are indicated by different lowercase letters for the same cultivar.

The effects of single and combined stresses on the phenotypic and growth parameters of the six alfalfa cultivars are shown in Figure 1. Both salt stress and combined salt and heat stress significantly decreased the plant height of all the alfalfa cultivars. However, heat stress significantly reduced the plant height of ‘Xinmu No. 4’, ‘Power 5030’, ‘Bara520YQ’, and ‘Barricade’ plants. Compared with the control and single stress treatments, combined stress had the greatest inhibition effect on plant height; the plant height of ‘Power 5030’ was 26.49% lower than that of the control, representing the greatest decrease among the six cultivars (Figure 1a,b). Under salt stress and combined stress, the branch numbers of all the cultivars were significantly lower than those of the control, except for ‘Xinmu No. 4’ and ‘Zhongmu No. 4’, whereas heat stress significantly increased the branch numbers of ‘WL358HQ’ and ‘Zhongmu No. 4’ (Figure 1c). The shoot fresh weight of all alfalfa cultivars under combined stress was significantly lower than that in the control and single stress treatments, except for ‘Xinmu No. 4’ under salt stress (Figure 1d). Regarding root fresh weight, the values for all cultivars were noticeably lower under combined stress than those of the control plants, with ‘WL358HQ’ exhibiting the greatest relative change (Figure 1e). In terms of shoot dry weight, the values for ‘Bara520YQ’, ‘WL358HQ’, and ‘Barricade’ under combined stress were significantly lower than those in the control and single stress treatments (Figure 1f). Compared with the other alfalfa cultivars, ‘Zhongmu No. 4’ presented the smallest relative changes in shoot fresh weight and dry weight under single and combined stresses (Figure 1d,f). Compared with those of the control plants, the total fresh biomass of all the alfalfa cultivars decreased by 5.45–25.53% and 1.9–23.98% under salt stress and heat stress, respectively, while it decreased by 28.22–39.97% under combined stress, with all cultivars showing significantly lower values than the control plants (Figure 1g). Specifically, ‘Power 5030’ and ‘Barricade’ exhibited significant decreases in biomass when subjected to single and combined stresses. Notably, under combined stress, the total fresh biomass of ‘Zhongmu No. 4’ decreased the least (28.22%), whereas that of ‘Barricade’ decreased the most (39.97%) (Figure 1g).

### 2.2. Effects of Single and Combined Stresses on Chlorophyll Content and Photosynthesis Characteristics

**Figure 2:**
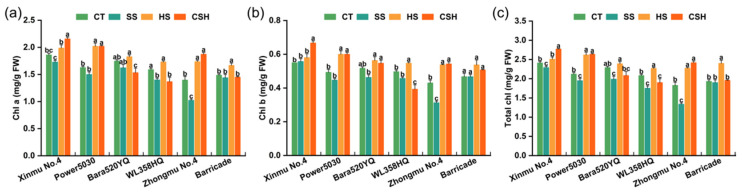
Changes in the chlorophyll contents of the six alfalfa cultivars under single and combined stresses. (**a**) Chlorophyll a, (**b**) chlorophyll b, and (**c**) total chlorophyll. The data are presented as the mean ± standard error (*n* = 3). Significant differences at the *p* < 0.05 level are indicated by different lowercase letters for the same cultivar.

Under combined stress, the Chl a and Chl b contents of ‘Xinmu No. 4’, ‘Power 5030’, and ‘Zhongmu No. 4’ plants were significantly higher than those of the control and salt stress-treated plants, while the Chl a and Chl b contents of ‘WL358HQ’ plants presented opposite trends, being significantly lower than those in the control and salt stress-treated plants (Figure 2a,b). All alfalfa cultivars showed a significant decrease in total chlorophyll content under salt stress compared with the plants in the control and heat stress treatments (with the exception of ‘Barricade’), whereas heat stress alone resulted in a greater total chlorophyll content in all cultivars than that in the control (except for ‘Xinmu No. 4’ and ‘Bara520YQ’). ‘Zhongmu No. 4’ exhibited the greatest decrease among the six cultivars under salt stress (Figure 2c). Under combined stress, the total chlorophyll contents of ‘Xinmu No. 4’, ‘Power 5030’, and ‘Zhongmu No. 4’ plants were significantly higher than those of plants in the control and salt stress treatments, whereas the total chlorophyll contents of ‘Bara520YQ’ and ‘Barricade’ plants were not significantly different from those in the control and salt stress treatments but were significantly lower than those under heat stress (Figure 2c).

**Figure 3:**
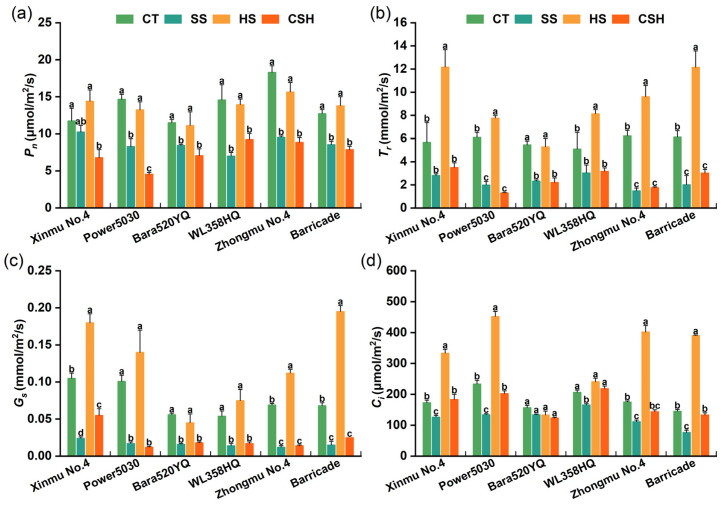
Changes in the photosynthetic parameters of different alfalfa cultivars under single and combined stresses. (**a**) Net photosynthetic rate, (**b**) transpiration rate, (**c**) stomatal conductance, and (**d**) intercellular CO_2_ concentration. The data are presented as the mean ± standard error (*n* = 5). Significant differences at the *p* < 0.05 level are indicated by different lowercase letters for the same cultivar.

Compared with those in the control and heat stress treatments, the *Pn* and *gs* significantly decreased under the salt and combined stress treatments, except for the *Pn* of ‘Xinmu No. 4’ under salt stress (Figure 3a,c). Under heat stress, the *Tr* of the six cultivars were significantly higher than those in the control, single salt stress, and combined stress treatments, except for ‘Bara520YQ’ in the control (Figure 3b,c). Compared with those of the control, the *Pn*, *Tr*, and *gs* of ‘Power 5030’ under combined stress decreased by 68.93%, 78.51%, and 88.12%, respectively (Figure 3a–c). The *Ci* of all the cultivars, except for ‘Bara520YQ’ and ‘WL358HQ’, were significantly higher under heat stress than in the control, salt stress, and combined stress treatments (Figure 3d). Compared with that of the control plants, the *Ci* of the ‘Bara520YQ’ plants did not significantly change under either single stress or combined stress treatment (Figure 3d).

### 2.3. Effects of Single and Combined Stresses on Na^+^ and K^+^ Contents

**Figure 4:**
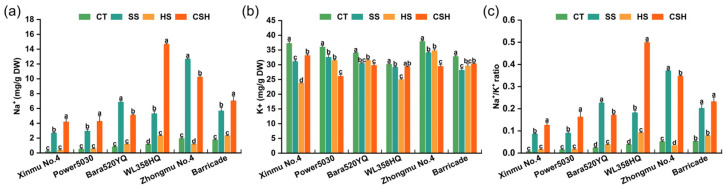
Changes in the leaf (**a**) Na^+^ concentration, (**b**) K^+^ concentration, and (**c**) Na^+^/K^+^ ratio of different alfalfa cultivars under single and combined stresses. The data are presented as the mean ± standard error (*n* = 3). Significant differences at the *p* < 0.05 level are indicated by different lowercase letters for the same cultivar.

The leaf Na^+^ content increased significantly under salt and combined stresses compared with that in the control and heat stress treatments, and the leaf Na^+^ content under combined stress was significantly higher than that in plants under salt stress only, except for ‘Bara520YQ’ and ‘Zhongmu No. 4’ (Figure 4a). In contrast, the K^+^ content significantly decreased in all cultivars under the three stress treatments compared with the control, except for ‘WL358HQ’ under combined stress (Figure 4b), resulting in a noticeably increased Na^+^/K^+^ ratio for all cultivars (Figure 4c). Among the six cultivars under combined stress, the Na^+^ contents of ‘Xinmu No. 4’ and ‘WL358HQ’ plants were 20.45 times and 12.74 times higher, respectively, than those of the control. Among the six cultivars, the K^+^ concentration of ‘Power 5030’ and ‘Zhongmu No. 4’ plants decreased the most, by 27.58% and 22.27%, respectively, compared to the control.

### 2.4. Effects of Single and Combined Stresses on Leaf Electrolyte Leakage, Relative Water Content, and Osmoregulatory Substance Content

**Figure 5:**
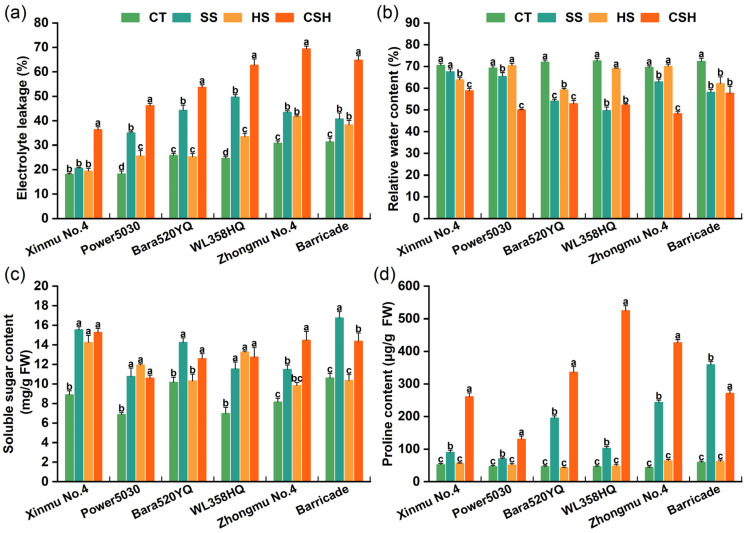
Changes in (**a**) electrolyte leakage, (**b**) relative water content, (**c**) soluble sugar content, and (**d**) proline content of different alfalfa cultivars under single and combined stresses. The data are presented as the mean ± standard error (*n* = 3). Significant differences at the *p* < 0.05 level are indicated by different lowercase letters for the same cultivar.

Under combined stress, the leaf RWC was significantly lower and the leaf EL was significantly higher in all the cultivars than in the control (Figure 5a,b). Among the six cultivars, the ‘WL358HQ’ plants showed the largest increase in leaf EL (154.09%), while the ‘Zhongmu No. 4’ plants showed the lowest leaf RWC (30.68%) under combined stress. Both the EL and RWC of leaves were significantly affected in three alfalfa cultivars (‘Power 5030’, ‘WL358HQ’, and ‘Zhongmu No. 4’) under salt stress compared to those of the control plants. The RWC and EL of ‘Barricade’ were significantly affected under the three stress conditions compared to those of the control plants.

Compared with the control, the salt and combined stress treatments significantly increased the leaf Pro content and SS content in all the cultivars. However, heat stress alone resulted in a greater SS content in the three cultivars than in the control (‘Xinmu No. 4’, ‘Power 5030’, and ‘WL358HQ’). The Pro content did not significantly change under heat stress in any cultivar compared with the control (Figure 5c,d). Under combined stress, the Pro content of all cultivars was significantly higher than that under salt stress (except for ‘Barricade’ plants), and the Pro contents of ‘WL358HQ’ and ‘Zhongmu No. 4’ were 11.26 and 9.79 times greater than that of the control, respectively, representing the greatest increases among the six cultivars (Figure 5d).

### 2.5. Effects of Single and Combined Stresses on ROS Accumulation and Antioxidative Enzyme Activity

**Figure 6:**
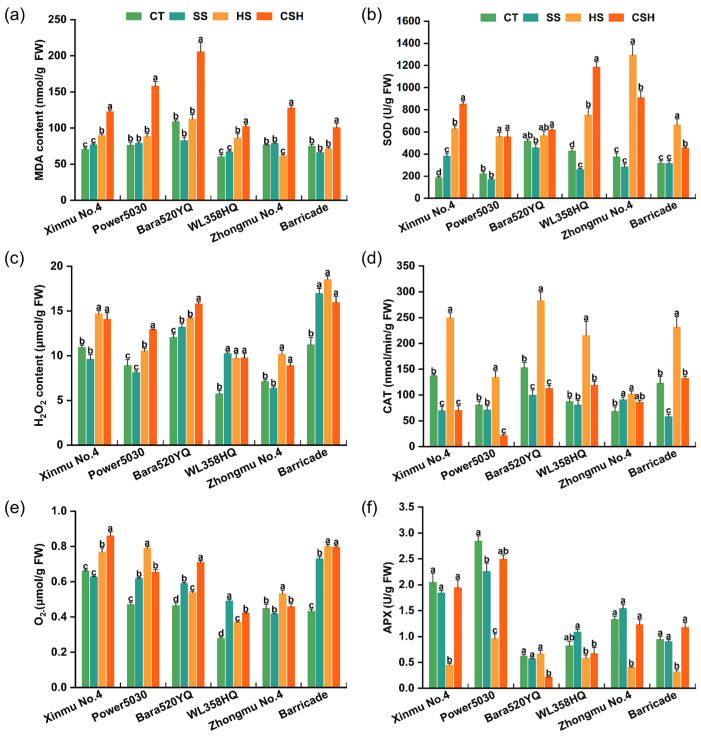
Changes in malondialdehyde (MDA) content (**a**), ROS content (**c**,**e**), and the activities of SOD (**b**), CAT (**d**), and APX (**f**) in different alfalfa cultivars under single and combined stresses. The data are presented as the mean ± standard error (*n* = 3). Significant differences at the *p* < 0.05 level are indicated by different lowercase letters for the same cultivar.

The leaf MDA content increased significantly under combined stress compared with that in the control and single stress treatments in all cultivars, indicating elevated membrane lipid peroxidation. The MDA contents of ‘Power 5030’ and ‘Bara520YQ’ under combined stress were 2.08 and 1.89 times greater than that of the control (Figure 6a). In terms of ROS accumulation, under salt stress, the H_2_O_2_ and O_2_^−^ contents of ‘Bara520YQ’, ‘WL358HQ’, and ‘Barricade’ plants were significantly higher than those of the control, whereas the H_2_O_2_ and O_2_^−^ contents of ‘Xinmu No. 4’ and ‘Zhongmu No. 4’ plants were not significantly different from those of the control (Figure 6c,e). Compared with the control, heat and combined stress significantly increased the contents of H_2_O_2_ and O_2_^−^ in all cultivars, except for O_2_^−^ in ‘Zhongmu No. 4’ under combined stress. The H_2_O_2_ and O_2_^−^ contents of ‘Barricade’ plants presented the greatest increase among all cultivars under both single and combined stresses (Figure 6c,e).

Heat and combined stress significantly induced leaf SOD activity in all alfalfa cultivars compared with those in the control and salt stress treatments, except for ‘Bara520YQ’. SOD activity was significantly higher in ‘Xinmu No. 4’ plants and significantly lower in ‘WL358HQ’ plants under individual salt stress than in the control (Figure 6b). Under heat stress, CAT activity significantly increased in all cultivars compared with that in the control, salt, and combined stress treatments, except for ‘Zhongmu No. 4’. Compared with that of the control, the CAT activity of ‘Xinmu No. 4’ and ‘Bara520YQ’ significantly decreased under the salt and combined stress treatments (Figure 6d). Compared with the control, heat stress significantly decreased APX activity in four alfalfa cultivars (‘Xinmu No. 4’, ‘Power 5030’, ‘Zhongmu No. 4’, and ‘Barricade’). The APX activities of ‘Power 5030’ under salt stress and ‘Bara520YQ’ under combined stress were significantly lower than those of the control plants (Figure 6f).

### 2.6. Multivariate Analyses

**Figure 7:**
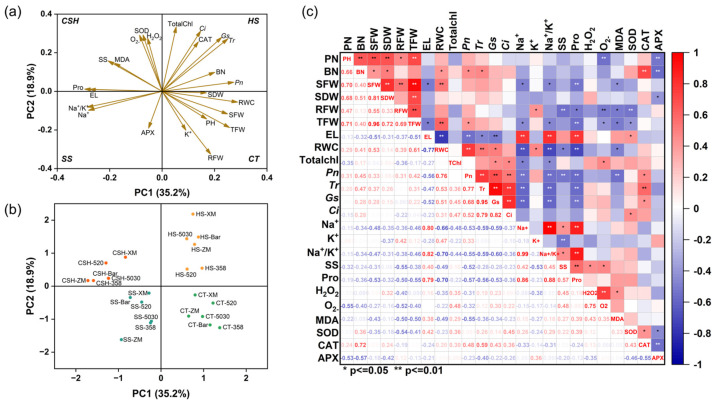
Multivariate analyses of alfalfa responses to single and combined stresses. (**a**) Principal component analysis (PCA) of morpho−physiological and biochemical indicators under salt, heat, and combined stresses. (**b**) PCA of six alfalfa cultivars under different stress conditions. (**c**) Pearson’s correlation analysis of different response variables. (**a**) CT = control; SS = salt stress; HS = heat stress; and CSH = combined salt and heat stresses. (**b**) CT−XM, CT−520, CT−ZM, CT−5030, CT−Bar, and CT−358 indicate alfalfa cultivars of ‘Xinmu No. 4’, ‘Bara520YQ’, ‘Zhongmu No. 4’, ‘Power 5030’, ‘Barricade’, and ‘WL358HQ’ under the control condition, respectively. The same prefix coding applies to stress treatments (e.g., SS−XM for ‘Xinmu No. 4’ under salt stress). (**c**) ** and * represent extremely significant correlation (*p* < 0.01) and significant correlation (*p* < 0.05), respectively.

Correlations between parameters and alfalfa cultivars under heat, salt, and combined stress were determined using PCA and Pearson’s correlation (Figure 7). The first four principal components in the PCA contributed 35.2%, 18.9%, 13.8%, and 10.5% of the total variability, respectively. Although some similarity was observed between the different stress treatments, the PCA results separated the four treatments into four quadrants. Heat stress was in the first quadrant, with the clustered variables being primarily chlorophyll and photosynthesis characteristics, which indicated that these variables were the key physiological indicators of the alfalfa response to heat stress. Combined stress was in the second quadrant, with the clustered variables including ROS, MDA, SS, Pro, and the SOD enzyme. Salt stress was in the third quadrant, and the clustered variables were EL, Na^+^, the Na^+^/K^+^ ratio, and the APX enzyme. In the fourth quadrant, the clustering variables mainly included growth parameters, K^+^, and RWC, which were all positive indicators under the control treatment. The data indicated that different parameters were impacted by the different stress treatments (Figure 7a,b). In addition, there were significant differences in the responses of different alfalfa cultivars to salt and heat stresses (Figure 7b). The six alfalfa cultivars were scattered around the positive *x*-axis for the control and heat treatments and the negative *x*-axis for the salt and combined stress treatments. Pearson’s correlation analysis revealed correlations between all response parameters. Among them, the biomass and photosynthesis characteristics of alfalfa were significantly negatively correlated with the Na^+^ concentration and membrane peroxidation indicators; the Pro content was significantly positively correlated with EL and the Na^+^ concentration; and the leaf RWC was significantly positively correlated with BN, SFW, TFW, *Pn*, *Tr*, *gs*, and the K^+^ concentration, whereas it was significantly negatively correlated with EL, the Na^+^ concentration, the Na^+^/K^+^ ratio, and Pro and MDA contents (Figure 7c).

### 2.7. Clustered Heatmap Analyses

**Figure 8:**
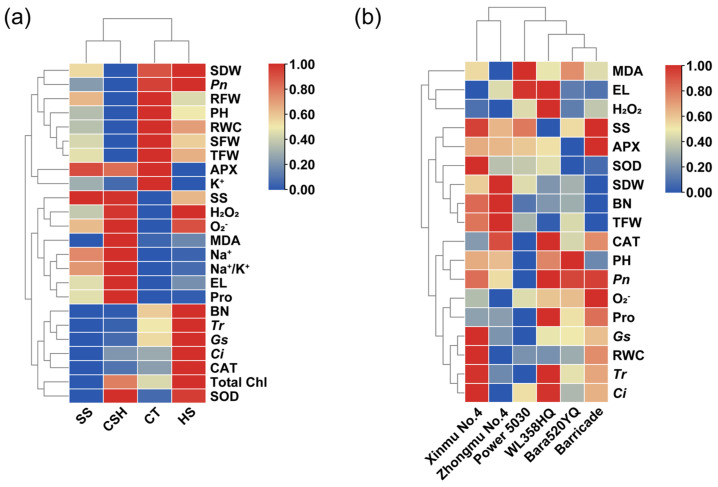
Cluster analysis of agronomic traits with physiological and biochemical indicators of six alfalfa cultivars under single stress and combined stress conditions. (**a**) Changes in agronomic and physiological traits under four treatments. (**b**) Changes in agronomic and physiological traits of six alfalfa cultivars under combined stress treatment.

Cluster analyses were carried out on 24 dependent variables under salt, heat, and combined stress treatments (Figure 8a), as well as on 18 dependent variables of the six alfalfa cultivars under combined stress treatment (Figure 8b), based on the variability of agronomic traits, photosynthetic characteristics, and physiological and biochemical indicators of different alfalfa cultivars. As shown in Figure 8a, salt stress and combined stress data were grouped together, while control and heat stress data were grouped together in another category. The color changes in the heatmap showed that agronomic traits, such as the plant height and biomass, as well as eight indices, such as the RWC, *Pn*, and K^+^ concentrations, were numerically lower under combined stress conditions than in the control and single stresses. In contrast, seven physiological indicators, namely, the SS, Pro and MDA contents, EL, and the H_2_O_2_, O_2_^−^, and Na^+^ concentrations were all higher under combined stress than in the control. Notably, the number of branches, photosynthetic performance (*Tr*, *gs*, and *Ci*), total chlorophyll content, and CAT activity of alfalfa under heat stress were higher than in other treatments. In addition, both combined stress and heat stress caused simultaneous increases in the H_2_O_2_ and O_2_^−^ concentrations, SOD activity, and total chlorophyll content, which were higher than those in the control and salt stress treatments (Figure 8a).

Cluster analysis results of the six alfalfa cultivars under combined stress are shown in Figure 8b. ‘Xinmu No. 4’ and ‘Zhongmu No. 4’ were clustered into one cluster, and the clustering characteristics were relatively high or medium values of alfalfa yield-related indices (SDW, BN, and TFW) and antioxidant enzyme activities (APX and SOD), while the values of membrane lipid peroxidation (MDA and EL) and reactive oxygen species (H_2_O_2_ and O_2_^−^) indicators were low. Furthermore, the photosynthetic performance indicators, such as the *Tr*, *gs*, and *Ci*, and leaf RWC values of ‘Xinmu No. 4’, were the highest. Compared with ‘Xinmu No. 4’, ‘Power 5030’ had the highest values of membrane lipid peroxidation (MDA and EL), but the lowest values of antioxidant enzyme activities (SOD and CAT), photosynthetic performance, and agronomic traits (PH, BN, SDW, and TFW). ‘WL358HQ’, ‘Bara520YQ’, and ‘Barricade’ had similar clustering characteristics, which showed that the values of photosynthetic performance indicators, such as *Pn*, *Tr*, and *gs*, the Pro content, and CAT activities were relatively high or medium, while the accumulation of MDA and O_2_^−^ was obvious, and the values of yield-related indicators (SDW, BN, and TFW) were low.

## 3. Discussion

### 3.1. Analysis of Morphology and Growth Parameters in Alfalfa in Response to Single and Combined Stresses

Under natural conditions, salinity usually occurs in the form of progressive chronic stress [31], which reduces plant productivity by affecting cell growth and metabolic processes, causing irreversible damage to seed germination, seedling growth, and crop yield [13,32]. Crops affected by salinization are often subjected to heat stress, which is caused by the combination of salinity and heat, especially in arid or semiarid regions [26]. In this study, single stresses and the combination of salt and heat stress affected the accumulation of aboveground biomass and the growth of roots of alfalfa to varying degrees, but the growth inhibition effect of combined stress was significantly greater than that of single stresses. Specifically, combined stress significantly affected plant morphology and decreased the plant height, shoot biomass per plant, and root fresh weight to a greater extent than single stresses. The synergistic effect of salt and high temperature leads to plant dwarfism and reduced yield [33], which is due to increased ionic toxicity and the reduction in photosynthetic energy acquisition (due to decreased photosynthesis and an increased Na^+^/K^+^ ratio) under combined stress conditions. Plants need to redistribute energy from growth and development to defense pathways against oxidative stress and sodium toxicity, which ultimately decreases yield [31,34]. Furthermore, we found that the alfalfa cultivars presented significant differences in tolerance to various stress treatments. For instance, both ‘Xinmu No. 4’ and ‘Zhongmu No. 4’ plants showed minimal growth inhibition, and their biomass decreased under the three stress treatments, indicating excellent stress tolerance. This stress tolerance trait has also been observed in other crops. Wheat varieties that tolerate combined salt and drought stress usually have more developed root structures [35], while quinoa varieties with high tolerance can maintain higher biomass accumulation and grain yield [23].

### 3.2. Analysis of Chlorophyll and Photosynthesis Characteristics in Alfalfa in Response to Single and Combined Stresses

Chlorophyll synthesis and photosynthesis in plants are highly sensitive to combined stress. The degree of damage to the plant photosynthetic system caused by combined salt–heat, salt–drought, or salt–heat–drought stress is much higher than that caused by single stresses [9,20,31]. The mechanism involves multiple factors: osmotic stress and ion toxicity cause cellular metabolic disorders, leading to stomatal closure and a decreased photosynthetic rate in plants [35,36], whereas high temperatures directly damage the structural integrity of photosystem II, shorten the crop growth cycle, and eventually prevent the growth and development of plants [37,38]. Rodrigues et al. [20] confirmed that salt stress and a combination of salt and heat stresses significantly inhibited chlorophyll synthesis and photosynthesis in tomato, while Zhou et al. [5] found that chlorophyll synthesis in heat-tolerant tomato varieties increased under heat stress. Similarly, our research revealed that the total chlorophyll content of five alfalfa cultivars was considerably decreased by salt stress alone, whereas heat stress significantly affected the accumulation of chlorophyll in four alfalfa cultivars. The cultivars significantly affected by heat stress (‘Xinmu No. 4’, ‘Power 5030’, and ‘Zhongmu No. 4’) presented the same accumulation trend in terms of chlorophyll content under combined stress. These results indicate that chlorophyll has specific response patterns and differences in type among different stress types and alfalfa cultivars.

With respect to the photosynthetic parameters of alfalfa, we found that the *Tr* and *gs* of alfalfa leaves increased under heat stress, while salinity stress inhibited *Tr*, *gs*, and *Pn*, indicating that the effect mechanisms of salt and high temperature on photosynthesis are fundamentally different. However, the inhibition of the alfalfa photosynthetic system by combined salt and heat stress followed a similar response pattern to that of salt stress. Similar phenomena have also been reported in studies of the response of soybean to combined salt and drought stress and the response of tomato to combined salt and heat stress [10,25,39]. Thus, we conclude that salt stress might play a predominant role in regulating the photosynthetic physiology of alfalfa in response to combined salt and heat stress. This specific regulatory mechanism significantly reduces biomass accumulation by inhibiting photosynthesis. Notably, the branch number, *Pn*, and *Tr* of ‘Xinmu No. 4’ were not significantly affected by salinity stress, indicating that this variety may maintain nearly normal photosynthesis through a unique salt tolerance mechanism.

### 3.3. Analysis of K^+^ and Na^+^ Levels in Alfalfa in Response to Single and Combined Stresses

Ion concentration analysis indicated that all alfalfa cultivars exhibited significant Na^+^ accumulation and K^+^ suppression in the leaves, suggesting that salinity stress disrupted the ion homeostasis of the plants and induced Na^+^ toxicity. However, we found that the Na^+^ concentration accumulated in the leaves of different alfalfa cultivars exhibited significant differences under salt and combined stresses. For example, ‘Bara520YQ’ and ‘Zhongmu No. 4’ accumulated much less Na^+^ under combined stress than under single salt stress, whereas the other four alfalfa cultivars accumulated significantly more Na^+^ under combined stress. Similar phenomena have been observed in studies of different tomato genotypes [25], which might be due to differences in root absorption, transport, and isolation of Na^+^ under different stress treatments [26,40], as well as the unique Na^+^ absorption regulatory mechanisms of different alfalfa cultivars. In addition, both single stress and combined stress significantly inhibited the concentration of K^+^. Unlike the mechanism by which salt stress and combined stress indirectly inhibit K^+^ absorption via Na^+^ accumulation, we found that heat stress directly weakens the K^+^ absorption capacity of alfalfa. All alfalfa cultivars showed a significantly reduced K^+^ content in leaves under heat stress compared with the control plants. This may be due to high temperatures disrupting the stability of the plasma membrane, resulting in increased membrane permeability and altered fluidity, leading to ion efflux [38]. The Na^+^/K^+^ ratio is a key indicator of normal cellular metabolism and cellular damage [15]. In this study, the Na^+^/K^+^ ratio under salt stress and combined stress was dramatically elevated by the increase in Na^+^ and the decrease in K^+^ in alfalfa leaves. These findings suggest that cellular metabolism and osmotic balance may be disrupted in plant leaves. Moreover, the strong negative correlations of Na^+^ with alfalfa biomass, chlorophyll synthesis, and photosynthesis parameters suggest that salt stress and combined salt and heat stress inhibit plant growth, development, and photosynthesis by disrupting ionic homeostasis.

### 3.4. Physiological Response of Alfalfa to Single and Combined Stresses

As key osmoregulatory substances, SS and Pro play crucial roles in the ability of plants to cope with adverse conditions [32]. We found that both salt stress and combined salt and heat stress induced the accumulation of SS and Pro in alfalfa. However, the accumulation of osmoregulatory substances under heat stress was unique. Heat stress did not significantly promote Pro synthesis, and SS significantly accumulated only in ‘Xinmu No. 4’, ‘Power 5030’, and ‘WL358HQ’ plants. This result confirms the view that different plant species selectively accumulate different osmoregulatory substances depending on the type of stress [26]. Furthermore, the amount of Pro synthesized in response to combined stress is significantly higher than that in response to salt stress alone, which is similar to the findings of previous studies on combined stresses such as cold–salinity [41], salt–drought [42], and salt–heat–light [43], indicating that the quantity of Pro accumulated is closely related to stress intensity.

Different stress conditions can trigger lipid peroxidation reactions in plant cell membranes, resulting in structural damage to the cell membranes. The EL and MDA contents can reflect the degree of damage to the cell membrane system caused by stress [44]. In this study, the contents of EL and MDA in alfalfa leaves under combined stress were significantly higher than those in the control and single stress treatments, indicating that combined stress exacerbated the degree of membrane lipid peroxidation through synergistic effects, causing severe membrane structure damage. In addition, EL and MDA were significantly negatively correlated with the total fresh biomass, leaf RWC, and *Pn* of alfalfa, indicating that oxidative damage induced by either single or combined stress significantly affects plant biomass and photosynthesis.

In the plant response to abiotic stress, ROS act as early stress signaling molecules that can trigger defense responses. However, increases in stress duration and intensity can disrupt photosynthesis and enhance photorespiration, leading to an imbalance in cellular redox homeostasis and excessive accumulation of ROS [45]. This study revealed that salt, heat, and combined stress induced the generation of ROS (H_2_O_2_ and O_2_^−^) in alfalfa, with higher ROS accumulation under heat stress and combined stress. Considering the changes in EL and MDA contents, these findings indicate that alfalfa suffers the most severe oxidative damage under combined salt and heat stress, which is consistent with the response patterns of other plants under combined stress [21,41,42,46]. Notably, the EL, MDA, and ROS contents of ‘Xinmu No. 4’ plants under salt stress did not significantly differ from those of the control plants. This result indicates that this variety possesses a unique salt tolerance mechanism that can effectively maintain the integrity of the cell membrane structure and avoid oxidative damage induced by salinity stress.

Plants alleviate ROS-induced oxidative damage through a synergistic system composed of antioxidant substances and antioxidant enzymes [47,48]. This study showed that ROS accumulation caused by heat stress can significantly increase the activities of SOD and CAT. However, when heat and salt stress occur in combination, although the activity of SOD still maintains an upward trend, the activities of CAT and APX show a downward trend in some alfalfa cultivars, indicating that combined stress can disrupt the synergistic effect of the antioxidant enzyme system, ultimately leading to oxidative stress. Furthermore, we found that the APX activity of alfalfa showed a significant selective response to heat stress. All alfalfa cultivars showed considerably lower APX activity under heat stress than in the control, salt stress, and combination stress treatments, with the exception of ‘Bara520YQ’ and ‘WL358HQ’, which was in line with previous findings in tomato [25], indicating that APX activity is affected by the type of stress and the characteristics of the alfalfa cultivars.

### 3.5. Multivariate Analysis and a Clustered Heatmap of Alfalfa Responses to Single and Combined Stresses

This study revealed the interrelationships between alfalfa cultivars, stress types, and variables through Pearson’s correlation analysis, PCA, and clustered heatmap analysis, which can be used to analyze covariate relationships and correlations among multiple variables [23,49]. The results of PCA and cluster analysis revealed that alfalfa plants exhibited significant photosynthetic and physiological adaptive responses under heat stress. Specifically, the photosynthesis efficiency was significantly improved and was accompanied by increases in the stomatal conductance and transpiration rate, which effectively reduced the leaf temperature, which was crucial for plants to improve their thermotolerance [5]. Moreover, chlorophyll synthesis significantly increased under heat stress, and these physiological adaptation changes might indirectly promote the number of alfalfa branches. The combination of salt and heat stresses triggered an obvious oxidative stress response, manifested as membrane damage caused by the accumulation of ROS, while simultaneously triggering the synthesis of osmotic regulatory substances (SS, Pro) and activating the SOD defense mechanism [50]. The significant accumulation of Na^+^ under salt stress resulted in an increase in EL and substantial Pro synthesis. There were significant positive correlations between EL, MDA, Pro, SS, H_2_O_2_, O_2_^−^, and the Na^+^/K^+^ ratio under salt stress and combined stress, and all were negatively correlated with growth/photosynthesis parameters, indicating that ion imbalance (Na^+^ accumulation) causes membrane damage and influences osmotic regulation, ultimately inhibiting photosynthesis and the growth and development of alfalfa. In addition, the correlation analysis of the variables in the control group showed a significant positive correlation between various agronomic traits, indicating a high degree of synergy between biomass accumulation and the morphological development of alfalfa under normal growth conditions.

Cluster heatmap analysis revealed the differences between agronomic traits and physiological responses of different alfalfa cultivars under combined stress conditions. Heatmap analysis demonstrated that the ‘Xinmu No. 4’ and ‘Zhongmu No. 4’ cultivars exhibited outstanding performance in both fresh and dry biomass accumulation, as well as ROS scavenging abilities, showing excellent adaptability to combined stress. Further analysis indicated that ‘Xinmu No. 4’ had significant photosynthetic advantages, maintaining relatively high photosynthetic efficiency even under combined stress conditions. In contrast, ‘Power 5030’ was sensitive to combined stress, which was manifested by severe membrane lipid peroxidation damage, and significantly decreased photosynthetic performance and key agronomic traits. These results clearly demonstrate significant varietal differences in the adaptive capacity of alfalfa to combined stress conditions. However, although it is clearly that the alfalfa cultivar ‘Xinmu No. 4’ exhibits tolerance under short-term combined stress, due to the diversity of external environmental conditions and the fact that plant responses to abiotic stress are regulated by multiple factors, whether this cultivar can still maintain excellent tolerance to combined stress under long-term stress conditions still needs further verification.

## 4. Materials and Methods

### 4.1. Plant Materials and Treatment

Table 1 lists the specifics of the six alfalfa cultivars (*Medicago sativa* L.) that were used in this study. Uniform-sized seeds of the six cultivars were surface-sterilized with 75% alcohol solution for 1 min, rinsed with ultrapure water 6 times, and then placed in Petri dishes and transferred to an artificial climate chamber (RXZ-160B, Jiangnan, China) at 25/20 °C under a 14 h/10 h (day/night) cycle and 50% relative humidity for germination. After the seeds sprouted, they were individually planted in seedling pots (7 cm length × 7 cm width × 7.8 cm height) with a mixed substrate (peat: vermiculite: perlite = 3:1:1, *v*/*v*/*v*) and placed in a greenhouse for growth. The conditions in the greenhouse were as follows: a photosynthetic photon flux density (PPFD) of 300 µmol m^−2^ s^−1^ with temperatures of 25/20 °C (day/night) and relative humidity between 50 and 70%. The pots were watered with ultrapure water every 3 days and half-Hoagland nutrient solution once a week.

After four weeks of cultivation, uniform and healthy seedlings were transferred to an artificial climate chamber for acclimation for 3 days, with 50–70% relative humidity and a photoperiod of 14 h/10 h (day/night) at a PPFD of 300 µmol m^−2^ s^−1^. Then, a total of 180 uniform-size alfalfa seedlings per cultivar were divided into four groups and treated in a completely randomized design as follows: (1) control (CT): irrigated with 60 mL ultrapure water per pot every two days and grown at 25/20 °C (day/night); (2) salt stress (SS): irrigated with 60 mL of 200 mM NaCl solution per pot every two days and grown at 25/20 °C (day/night); (3) heat stress (HS): irrigated with 60 mL ultrapure water per pot every two days and grown at 38/28 °C (day/night); and (4) combined salt and heat stress (CSH): irrigated with 60 mL of 200 mM NaCl solution per pot every two days and grown at 38/28 °C (day/night). The concentration of NaCl used in the treatments was determined based on previous reports [51,52]. After 7 days of stress treatment, morphological parameters were measured, and plant leaf samples were collected. The samples were immediately frozen in liquid nitrogen and then stored at −80 °C. The experiment was repeated 3 times.

### 4.2. Agronomic Traits and Physio-Biochemistry Analysis

Plant height, number of branches, shoot fresh and dry weights, root fresh weight, and total fresh biomass were measured after 7 days of treatment with ten replications. The 3rd and 4th fully expanded leaves from the top of the alfalfa plants were used to determine the physio-biochemistry parameters after 7 days of four treatments. There were three independent biological replications with two technical replicates for the measurements.

Leaf electrolyte leakage (EL) was measured according to the method of Wu et al. [53] with slight modifications. First, 0.1 g of alfalfa leaves was collected and washed with ultrapure water. The leaves were cut into small strips, immersed in test tubes containing 25 mL of ultrapure water, agitated for 30 min, and incubated at 25 °C for 6 h. The conductivity was measured using a DDSJ-308F conductivity tester (LeiCi, Shanghai, China), and the values were recorded as EL1. Then, the centrifuge tubes were placed in boiling water for 30 min and cooled to room temperature, and the values were recorded as EL2. Finally, EL (%) was calculated as EL1/EL2 × 100.

Leaf relative water content (RWC) was determined according to the method of Zhang et al. [54] with a slight modification. In brief, 0.1 g of fresh leaf samples was weighed, placed in a centrifuge tube containing ultrapure water, and soaked in the dark for 24 h. Then, the leaves were wiped dry with filter paper and weighed, and the values were recorded as DW. Afterward, the leaves were placed in an oven at 65 °C until the weight was stable, and the values were recorded as TW using the following equation: RWC (%) = (FW-DW)/(TW-DW) × 100.

Leaf ion concentrations were determined according to the method of Yang et al. [55] and Wei et al. [56] with some modifications. An amount of 0.1 g of dried leaf sample was weighed into a 125 mL beaker, 5 mL of sulfuric acid was added, and the sample was mixed and allowed to stand overnight. The flask was placed on an electric heating plate and heated slowly and evenly. Once white fumes were produced, 1 mL of 30% H_2_O_2_ was slowly added, and the solution was gently swirled. This step was repeated until the solution became clear. The liquid was filtered into a 50 mL volumetric flask and diluted to the mark with ultrapure water. The sample was further diluted on the basis of its ion content. The concentrations of K^+^ and Na^+^ ions were then measured using an atomic absorption spectrophotometer (240DUO, Agilent, Santa Clara, CA, USA).

Leaf antioxidant enzyme activities were determined according to the method of Sun et al. [57] with a slight modification. Fresh leaf samples (0.1 g) were homogenized on ice with 1 mL of prechilled phosphate buffer (pH 7.8) for the measurements of superoxide dismutase (SOD) and catalase (CAT), or with 1 mL of prechilled phosphate buffer (pH 7.0) containing 0.1 mM ascorbate and 1 mM EDTA for the measurement of ascorbate peroxidase (APX). After centrifugation at 12,000× *g* for 10 min at 4 °C, the supernatants were tested for the enzymes.

SOD and CAT activities were measured according to Hu et al. [58] with several modifications. The SOD reaction mixture contained 50 mM phosphate buffer (pH 7.8), 13 mM methionine, 75 mM nitrogen blue tetrazolium (NBT), 0.1 mM EDTA, and 2.0 µM riboflavin. An amount of 0.1 mL of enzyme extract was added to the reaction mixture solution and placed under 4000 lx fluorescent tubes for 15 min, and a blank control was set up for light− proof treatment. The absorbance was recorded at 560 nm. One unit of SOD activity was defined as the enzyme quantity causing 50% inhibition of NBT photoreduction.

The CAT reaction mixture consisted of 50 mM phosphate buffer (pH 7.0) and 100 mM H_2_O_2_. An amount of 190 µL of reaction solution was quickly mixed with 10 µL of enzyme extract, and the initial absorbance value at 240 nm and the absorbance value after 1 min were immediately recorded. The CAT activity unit was defined as the amount of enzyme required to catalyze the degradation of 1 nmol of H_2_O_2_ per minute.

The activity of APX was measured according to Shunkao et al. [59] with some modifications. The APX reaction mixture contained 50 mM phosphate buffer (pH 7.0), 0.3 mM ascorbate, and 0.06 mM H_2_O_2_. The reaction was initiated by 20 µL of enzyme extract, and the absorbance at 290 nm was recorded to determine the APX activity.

The contents of soluble sugar (SS), proline (Pro), malondialdehyde (MDA), hydrogen peroxide (H_2_O_2_), and the superoxide anion radical (O_2_^−^) in the leaf samples were determined by using micro assay kits (Comin Biotechnology Co., Ltd. Suzhou, Jiangsu, China) KY-1-Y, Pro-1-Y, MDA-1-Y, H_2_O_2_-1-Y, and SA-1-G, respectively. According to the kit instructions, 0.1 g of alfalfa leaves was weighed separately and mixed with 1 mL of the corresponding reagent. The mixture was ground into a homogenate on ice and then centrifuged. The supernatant was taken, and the corresponding reagent was added. The corresponding absorbance values were measured using UV spectrophotometry, and the contents of SS, Pro, MDA, H_2_O_2_, and O_2_^−^ were calculated based on the formulas provided in the instructions.

### 4.3. Chlorophyll Content and Photosynthetic Parameters

The chlorophyll (Chl) a and Chl b contents in the alfalfa leaves were determined as described by Zhang et al. [54]. In brief, 0.1 g of fresh leaf samples was weighed into test tubes containing 25 mL of acetone (80%, *v*/*v*) and soaked in the dark for 24 h. The absorbances of the samples were measured at 663 and 645 nm by using a spectrophotometer (T6, Persee, Beijing, China).

The 3rd and 4th leaves from the top of the alfalfa plants were used to measure the photosynthetic parameters with a Li-6400XT portable photosynthesis system (Li-COR, Lincoln, NE, USA), from 11:00 a.m. to 13:00 p.m., on the 8th day of treatments. The photosynthesis measurements were set to a PPFD of 500 µmol m^−2^ s^−1^, and the CO_2_ concentration was 400 µmol mol^−^^1^; the leaf temperature was set to 25 °C for the control and salt stress-treated plants and to 38 °C for the heat stress and combined stress-treated plants. The parameters included the transpiration rate (*Tr*), net photosynthetic rate (*Pn*), intercellular CO_2_ concentration (*Ci*), and stomatal conductance (*gs*). There were five biological replications per cultivar in each treatment.

### 4.4. Statistical Analysis

Excel 2021 and SPSS 26.0 software (SPSS Inc., Chicago, IL, USA) were used to statistically analyze the original data via analysis of variance (ANOVA), Pearson’s correlation analysis, and principal component analysis (PCA). The Shapiro–Wilk test and Bartlett’s test were used to evaluate the normality and homogeneity assumptions before analysis of variance was performed. Significant differences were calculated based on Duncan’s test at the *p* < 0.05 level. Cluster analyses of dependent variables for different stress treatments and different alfalfa cultivars were performed by using the TBtools v2.316 software. Figures were generated using the Origin 2024 and TBtools software.

## 5. Conclusions

This systematic analysis of the morphological and physiological traits of alfalfa indicated that the stress intensity caused by combined salt and heat stress in plants is significantly greater than that caused by single stresses. Combined stress severely disrupted the balance of ion homeostasis in alfalfa and exacerbated the degree of membrane peroxidation, ultimately inhibiting plant growth and development. These findings revealed that the cumulative effect of combined stress amplifies the negative effects of various stress factors on plants. Salinity plays a dominant role in regulating the photosynthetic physiological response of alfalfa to combined salt and heat stress. The reduction in photosynthetic efficiency under combined stress is closely associated with Na^+^ toxicity, ROS accumulation, and stomatal closure. This specific regulatory mechanism significantly reduces biomass accumulation by inhibiting photosynthesis. Among the tested varieties, ‘Xinmu No. 4’ exhibited excellent stress resistance with the smallest reduction in growth inhibition and well-maintained photosynthetic efficiency under different stress treatments. The damage to the membrane system and the accumulation of ROS were significantly lower in ‘Xinmu No. 4’ plants than in the other cultivars, indicating that this variety may effectively mitigate oxidative stress damage to photosynthesis by regulating redox balance, thereby maintaining a relatively stable biomass. The results provide a theoretical basis for the sustainable development of the grass industry in arid and saline–alkali soil regions and for the breeding of high-quality alfalfa varieties that are tolerant to combined salt and heat stress. In the subsequent research, we will focus on conducting long-term stress experiments to systematically evaluate the tolerance performance of the ‘Xinmu No. 4’ variety under prolonged stress periods, and integrate multi-omics technologies to deeply analyze the molecular response mechanism of alfalfa under combined salt and heat stress conditions.

## Figures and Tables

**Table 1:** Information on the alfalfa cultivars used in the research.

No.	Cultivars	Source
1	Xinmu No. 4	Harvest Forage Agro Co., Ltd. Urumqi, China.
2	Power5030	Harvest Forage Agro Co., Ltd. Urumqi, China.
3	Bara520YQ	Bailv (Tianjin) International Seed Co., Ltd. Tianjin, China.
4	WL358HQ	Beijing Rytway Seed Co., Ltd. Beijing, China.
5	Zhongmu No. 4	Institute of Animal Sciences of CAAS. Beijing, China.
6	Barricade	Beijing Green Animal Husbandry S&T Development Co., Ltd. Beijing, China.

## Data Availability

Data are contained within the article.
